# Improved Na^+^/K^+^ Storage Properties of ReSe_2_–Carbon Nanofibers Based on Graphene Modifications

**DOI:** 10.1007/s40820-019-0248-2

**Published:** 2019-03-11

**Authors:** Yusha Liao, Changmiao Chen, Dangui Yin, Yong Cai, Rensheng He, Ming Zhang

**Affiliations:** Key Laboratory for Micro/Nano Optoelectronic Devices of Ministry of Education, Hunan Provincial Key Laboratory of Low-Dimensional Structural Physics and Devices, School of Physics and Electronics, Hunan University, Changsha, 410082 People’s Republic of China

**Keywords:** Rhenium diselenide, Carbon nanofiber, Graphene, Sodium-/potassium-ion batteries, Full cell

## Abstract

Rhenium diselenide (ReSe_2_) has caused considerable concerns in the field of energy storage because the compound and its composites still suffer from low specific capacity and inferior cyclic stability. In this study, ReSe_2_ nanoparticles encapsulated in carbon nanofibers were synthesized successfully with simple electrospinning and heat treatment. It was found that graphene modifications could affect considerably the microstructure and electrochemical properties of ReSe_2_–carbon nanofibers. Accordingly, the modified compound maintained a capacity of 227 mAh g^−1^ after 500 cycles at 200 mA g^−1^ for Na^+^ storage, 230 mAh g^−1^ after 200 cycles at 200 mA g^−1^, 212 mAh g^−1^ after 150 cycles at 500 mA g^−1^ for K^+^ storage, which corresponded to the capacity retention ratios of 89%, 97%, and 86%, respectively. Even in Na^+^ full cells, its capacity was maintained to 82% after 200 cycles at 1C (117 mA g^−1^). The superior stability of ReSe_2_–carbon nanofibers benefitted from the extremely weak van der Waals interactions and large interlayer spacing of ReSe_2_, in association with the role of graphene-modified carbon nanofibers, in terms of the shortening of electron/ion transport paths and the improvement of structural support. This study may provide a new route for a broadened range of applications of other rhenium-based compounds.

**Electronic supplementary material:**

The online version of this article (10.1007/s40820-019-0248-2) contains supplementary material, which is available to authorized users.

## Highlights

- Graphene modifications effectively improved conductivity but also resulted in a regulatory effect on the decrease in its diameter.
- The synergistic action of graphene and carbon fibers protected the structure of the electrode material and shortened the ion diffusion path.
- ReSe_2_@G@CNFs exerted high capacity and long cyclic stability in Na^+^/K^+^ half cells. When this compound was assembled in Na^+^ full cells, the cells displayed excellent performances

**Electronic supplementary material:**

The online version of this article (10.1007/s40820-019-0248-2) contains supplementary material, which is available to authorized users.

## Graphical abstract

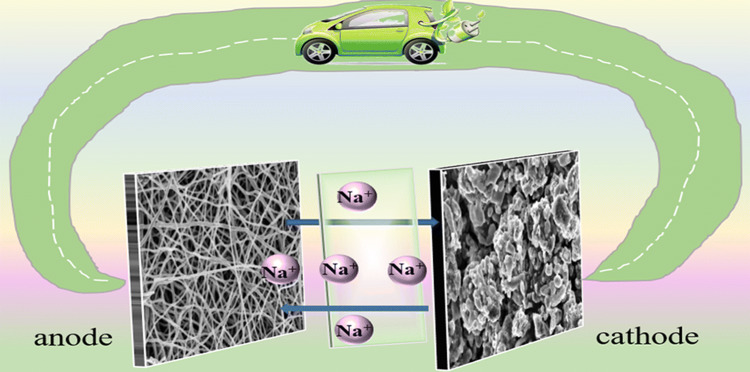

## Introduction

With the rapid development of electronic equipment and the emergence of electric and hybrid electric vehicles in recent years, it has become necessary to investigate energy storage materials with high efficiency, the existence of alternative and abundant resources, and the environment-friendliness [1–3]. Owing to the characteristics of the high energy density and broad voltage range, lithium-ion batteries (LIBs) have gained considerable attention since their development [4–7]. However, the limited distribution of lithium resources in the earth has hindered their widespread applications in grid energy storage system [8–10]. As promising alternatives to LIBs, sodium-ion batteries (NIBs) and potassium-ion batteries (KIBs) have attracted considerable interest owing to the abundance of sodium/potassium sources and their low prices [7, 11–14]. In addition, NIBs and KIBs have redox voltages which are closer to Na^+^/Na (− 2.71 V vs. normal hydrogen electrode) and K^+^/K (− 2.93 V) to that of Li^+^/Li (− 3.04 V) [15–17]. Nevertheless, Na^+^ (1.02 Å) and K^+^ (1.38 Å) have larger radii than Li^+^ (0.76 Å), which means that they are more likely to induce sluggish reaction kinetics and massive volume expansions, thus resulting in poor rate performances and unstable circulation [13, 18, 19]. Moreover, graphite has a capacity of 372 mAh g^−1^ in LIBs, exhibits a low capacity in KIBs (279 mAh g^−1^), but cannot be used in NIBs. Therefore, the need to identify electrode materials with larger physical spaces to adapt Na^+^/K^+^ fuel cells is imminent.

Transition metal dichalcogenides (TMDs) [20–22] are known for their excellent electrochemical properties as negative electrode materials. Rhenium diselenide (ReSe_2_) is a representative TMD and consists of a plane of Re atoms sandwiched between two planes of Se atoms. Every layer is coupled based on weak van der Waals interactions (18 MeV) owing to the extra valence electrons in Re atom [23]. Additionally, this particular compound has a larger interlayer spacing (6.37 Å) compared to other TMDs, such as ReS_2_ (6.14 Å) and MoS_2_ (6.15 Å). The larger distance between layers allows the insertion of Na^+^/K^+^ without significant destruction [24]. Secondly, based on acquired knowledge from most types of dichalcogenides arranged in hexagonal phases, ReSe_2_ is a distorted 1 − *T* phase with triclinic symmetry [25], thus possessing superior potential for strain engineering. And also, ReSe_2_ is an anisotropic semiconductor [26]. Results published by de Groot et al. [27] and Tiong et al. [28] showed that in terms of electrical conductivity, the orientation of the atomic link of Re–Re was better than those associated with other crystalline directions. However, the volume change during circulation will result in rapid capacity decline, and the conductivity of ReSe_2_ needs further improvements to better match the dynamics of the electrochemical reaction. Therefore, some efforts should be dedicated to engineer the composition and morphology of ReSe_2_.

As it is known, graphene exhibits tremendous potential and promise for the improvement of conductivity, cyclic stability, and control of the morphology of materials [29, 30]. Additionally, various porous carbon nanocomposites, including carbon nanofibers [31], carbon spheres [32], and carbon nanobelts [33], possess desirable characteristics given their capabilities for accommodating volume expansion, their large specific surface areas, and shortened ion or electron diffusion paths. To solve the aforementioned problems, in this study, we synthesized ReSe_2_–carbon nanofibers (termed as ReSe_2_@G@CNFs) with the use of electrospinning and solid-phase heat treatments. When these nanofibers are used as negative electrodes, they exhibit superb Na^+^/K^+^ storage performances. To research the effects of graphene and different rhenium contents on the properties of these materials, we also prepared ReSe_2_ composite nanofibers without graphene (termed as ReSe_2_@CNFs) and different concentrations of Re composite nanofibers. (The concentrations of rhenium are 1 and 0.4 mmol, and are thus denoted as 1 mM ReSe_2_@G@CNFs and 0.4 mM ReSe_2_@G@CNFs.)

## Experimental

### Material Synthesis

Ammonium perrhenate (NH_4_ReO_4_), poly(methyl methacrylate) (PMMA, Mw = 35,000), polyacrylonitrile (PAN, Mw = 150,000), and *N*,*N*-dimethylformamide (DMF) were used without further processing. Graphene oxide (GO) was obtained in accordance with a previously published document [34]. The ReSe_2_ composite nanofibers were synthesized by electrospinning and heat treatment as follows. Firstly, 0.7 mmol NH_4_ReO_4_ was added into 6 mL DMF and stirred for several minutes to completely dissolve. Small amounts of PAN and PMMA were then dissolved in the aforementioned solution with vigorous stirring at 50 °C in a water bath. Subsequently, 0.5 mL GO was added to form a light black solution and stirring was continued for another several hours. To prepare comparative carbon fibers, a precursor without GO and precursors containing 1 mmol/0.4 mmol NH_4_ReO_4_ were also prepared in the same way. All precursor solutions were poured into a 6-mL noncorrosive steel needle injector that was electrically connected to a high-voltage power supply (11–12 kV). The flow rate of the solution was controlled to an approximate value of 0.3 mL h^−1^. The distance between the needle and collector was set at to a value of 12 cm. After it was peroxided at 235 °C in air for 2 h, the as-spun reddish brown mats were annealed at 620 °C in argon for 2 h. Finally, the black membrane which was obtained was ground and mixed with selenium powder at the ratio of 1:1.5. The mixture was then placed into an ark and calcined in Ar/H_2_ at 270 °C for 3 h. The temperature was then increased to 600 °C and was maintained for 2 h. The ReSe_2_ composite fibers were obtained after this selenizing process.

### Material Characterization

The crystal structure of the as-prepared nanofibers was characterized using powder X-ray diffraction [XRD, Siemens D-5000 diffractometer with Cu–*K*_α_ irradiation (*λ* = 1.5406 Å)]. The microstructure of the samples was characterized using a HITACHI S4800 scanning electron microscope (SEM), a transmission electron microscope (TEM), and a high-resolution TEM operating at 200 kV. The samples were also analyzed by X-ray photoelectron spectroscopy (XPS, Surface Science Instruments S-probe spectrometer). The Raman spectrum was acquired at room temperature with excitation laser lines of 514 nm (Renishaw). Specific surface areas were measured using a Tristar II 3020 instrument by adsorption of nitrogen at 77 K. The pore diameter distribution of the mesopores was tested by nitrogen adsorption/desorption analysis (MicroActive ASAP 2460). The thermal gravimetric analysis was recorded on a thermogravimetric analyzer (TGA, PerkinElmer, Diamond TG/DTA) with a heating rate of 10 °C min^−1^ in air from 30 to 800 °C.

### Electrochemical Measurements

In a glove box with argon, the half cells were assembled using CR2025 coin cells. The anodes consisted of active materials, carbon black, and carboxymethyl cellulose (8:1:1). The mixed suspension was then coated on a copper foil and dried at 70 °C in a vacuum oven. The electrodes were cut into small round pieces with a diameter of 12 mm. The loading mass of active material was in the range of 1.1–1.5 mg cm^−2^ for the anode. Na or K metal was used as a counter electrode, and the glass fiber (Whatman, CF/F) was used as a separator to assemble NIBs and KIBs. The electrolyte was consisted of 1 M NaClO_4_ in ethylene carbonate (EC)/diethyl carbonate (DEC) (1:1) with 5% fluoroethylene carbonate (FEC) in the case of NIBs, and 3 M potassium bis(fluorosulfonyl)imide (KFSI) in methoxymethane (DME) in the case of KIBs, respectively. A Neware test system was used to conduct the galvanostatic charge–discharge measurements, within a voltage range of 0.01–3 V for anode materials, 2.0–3.9 V for the Na_3_V_2_(PO_4_)_3_ cathode, and 1–3.5 V for full cells. The calculation of the specific capacity was based upon the quality of the active substance of the entire electrode. The cyclic voltammetry (CV) curves at a scan rate of 0.1 mV s^−1^ and electrochemical impedance spectrum (EIS) were recorded using a CHI660E electrochemical workstation.

## Results and Discussion

**Fig. 1:**
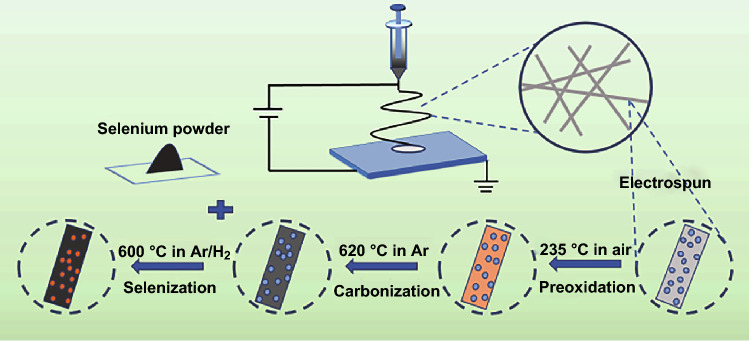
Schematic diagram of the preparation of ReSe_2_@G@CNFs

**Fig. 2:**
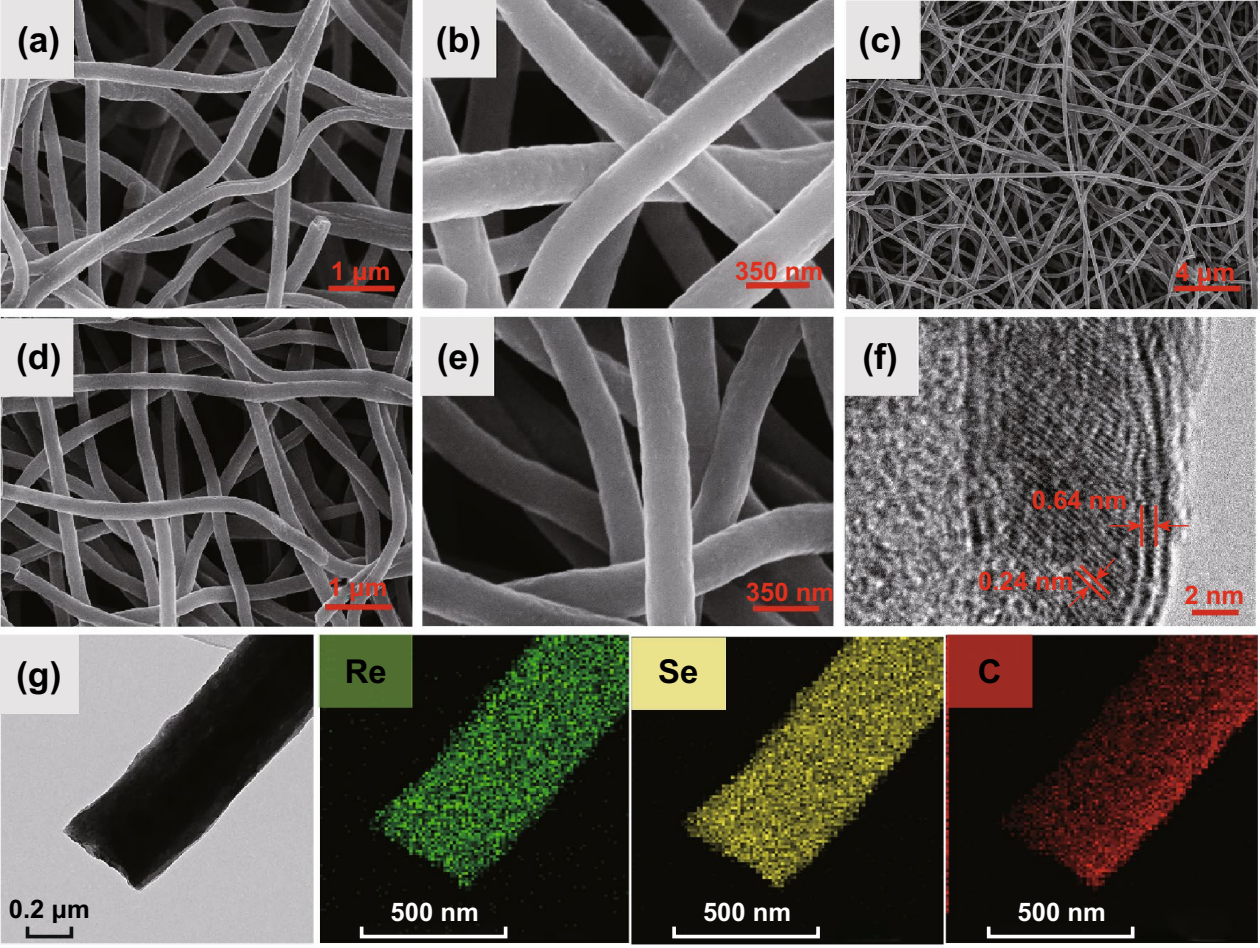
SEM images of **a**, **b** Re_2_O_7_@G@CNFs, and **c**–**e** ReSe_2_@G@CNFs. **f** HRTEM images of ReSe_2_@G@CNFs. **g** EDS mapping indicates the element distribution of the composites

A new method was meticulously explored to synthesize ReSe_2_@G@CNFs by electrospinning and heat treatment. The detailed process is schematically illustrated in Fig. 1. We first synthesized Re_2_O_7_ precursor carbon nanofibers by electrospinning. Subsequently, ReSe_2_@G@CNFs were prepared by mixing the precursor fibers and selenium powder and then annealed at high temperatures. The morphology of the composite was characterized by SEM and TEM. Figure 2a, b shows the SEM plots of the fibers annealed at 620 °C that have been shown to correspond to oxidized carbon nanofibers (Re_2_O_7_@G@CNFs) according to XRD (Fig. S1). Figure 2c–e shows the SEM images of the as-prepared ReSe_2_@G@CNFs. These fibers are uniform with an average diameter of approximately 244 nm and with a relatively smooth surface. There were no significant changes in diameters during selenization. Regarding the morphology of ReSe_2_@CNFs, the nanofiber surfaces without graphene were comparatively less smooth with an expanded diameter of approximately 308 nm, as displayed in Fig. S2. Based on statistical analyses, the diameters of the carbon fibers decreased following graphene modifications. This reflected the regulating effects of graphene on the material morphology, as shown in Fig. S3. Additionally, when the content of Re increased to 1 mmol, a large amount of Re metal was precipitated from the fiber surface after annealing treatment, as shown in Fig. S4a, b. Nevertheless, the particles disappeared and the fiber surface became smooth after selenization (Fig. S4c, d) owing to the decomposition of the excess amount of rhenium. As shown in Fig. S4e, f, ReSe_2_@G@CNFs (0.4 mM) with lowest rhenium contents had rougher surfaces and some noticeable wrinkles. The microstructures of ReSe_2_@G@CNFs were further characterized by TEM. In Fig. S5, the nanoparticles are scattered in the multihole channels owing to the synergy of carbon and graphene. The surfaces of the nanofibers are rough with pores, and few graphene sheets can be observed on the fiber surface. This is likely attributed to the low content of graphene and the fact that it is embedded in the carbon matrix [35, 36]. An HRTEM image (Fig. 2f) was also acquired to verify the phase of the nanoparticles. It displays the lattice space (dimension of 0.64 × 0.24 nm^2^), confirming the existence of the (100) and (102) lattice planes of ReSe_2_. The TEM element mapping of ReSe_2_@G@CNF in Fig. 2g shows that the Re, Se, and C are distributed uniformly throughout the carbon nanofibers.

**Fig. 3:**
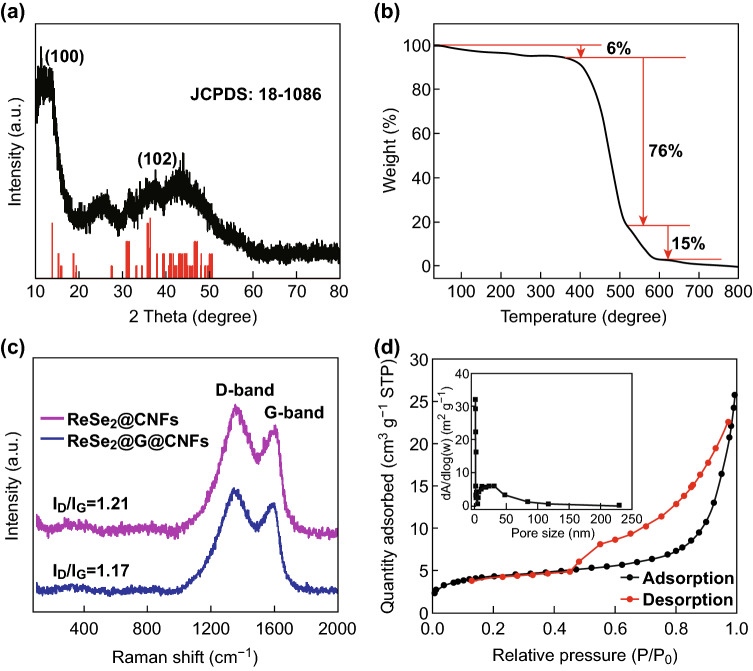
**a** XRD pattern, **b** TGA curve (in air). **c** Raman spectrum, and **d** nitrogen adsorption/desorption curve of the as-prepared ReSe_2_@G@CNFs. The inset shows the pore size distribution

The XRD results are shown in Fig. 3a. The diffraction peaks located at 13.8° and 36.3° correspond to the (100) and (102) triclinic lattice planes of ReSe_2_, respectively. It is also worth noting that the broad peak between 20° and 30° corresponds to the peak of the amorphous carbon. Almost all the main peaks can be indexed to ReSe_2_ (JCPDS No. 18-1086), thus suggesting that no other rhenium compounds were formed. TGA was performed to investigate the chemical composition of ReSe_2_@G@CNFs. As shown in Fig. 3b, the mass loss of 6% is mainly attributed to the loss of adsorbed solvent [37], which is related to the endothermic curve of differential thermal analysis shown in Fig. S6. As the temperature increases, a significant quality loss of 76% is observed. This is associated with a sharp exothermic peak in the temperature range of 360–510 °C and can be ascribed to the combustion of carbon nanofibers and graphene [38, 39]. Furthermore, the last stage is relevant to the oxidation and decomposition of ReSe_2_. This temperature is higher compared to the reported results on rhenium or selenium [40, 41], thus indicating the protective effect of carbon. These results provide evidence in support of the encapsulation of the ReSe_2_ nanoparticles in the carbon matrix, which are similar to the outcomes from TEM observations. Figure 3c shows the Raman spectra of two tested samples. The distinct peak located at 1358 cm^−1^ is known as D-band, and it originates from the A1g vibration pattern and is induced by defects and disorder. A G-band is detected at 1600 cm^−1^ and is produced by stretching all the *sp*^2^ atom pairs in a carbon ring or long chain [42]. The intensity ratio (*I*_D_/*I*_G_) is often used to measure the disorder of carbon materials. Additionally, the value of ReSe_2_@G@CNFs (*I*_D_/*I*_G_ = 1.17) is lower than that of ReSe_2_@CNFs (*I*_D_/*I*_G_ = 1.21), thus illustrating a higher degree of graphitization [43]. The specific surface of ReSe_2_@G@CNFs was characterized by nitrogen adsorption/desorption measurements. As shown in Fig. 3d, typical type IV nitrogen adsorption/desorption curves exert obvious H3 hystereses loops, thus indicating an evident mesoporous structure. The specific surface area of this sample is 15.72 m^2^ g^−1^, and the pore volume is 0.0038 cm^3^ g^−1^. Furthermore, most of pore sizes are less than 20 nm, as shown in the inset pore size distribution plot. Once again, these findings prove the existence of mesopores. XPS was also conducted to survey the chemical states of the sample. The survey spectrum in Fig. S7a shows that the major elements in ReSe_2_@G@CNFs are Re, Se, and C. Based on the high-resolution spectrum of C 1 s (Fig. S7b), only one large peak can be deconvoluted into two peaks at 284.8 and 286.2 eV, which can be, respectively, assigned to the C–C and C–O bonds. Figure S7c displays a high-resolution Re 4*f* spectrum of ReSe_2_@G@CNFs. The two peaks at 41.5 and 43.9 eV were classified as Re 4*f*_7/2_ and Re 4*f*_5/2_ [44], thereby indicating that rhenium does not contain any inherent oxide as metal species [24]. In a high-resolution Se 3*d* spectrum (Fig. S7d), the Se 3*d*_5/2_ and Se 3*d*_3/2_ peaks corresponded to the position of 54.7 and 55.6 eV [45].

**Fig. 4:**
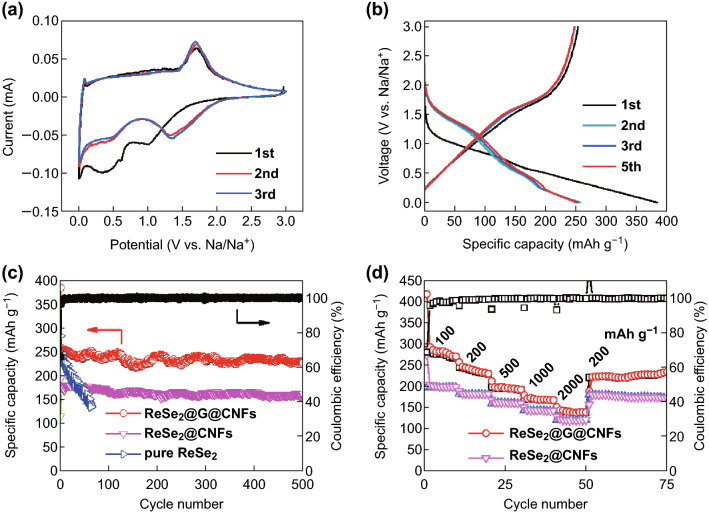
Electrochemical properties of NIBs. **a** CV curves of ReSe_2_@G@CNFs. **b** Charge and discharge graphs of ReSe_2_@G@CNFs at 200 mA g^−1^. **c** Long cyclic capability and corresponding CE of ReSe_2_@G@CNFs at 200 mA g^−1^ for 500 cycles. **d** Comparison of rate performance between ReSe_2_@G@CNFs and ReSe_2_@CNFs from 100 to 2000 mA g^−1^

The electrochemical properties of ReSe_2_@G@CNFs anode in the half cells of the Na ions are shown in Fig. 4. CV measurements within the voltage range of 0.01–3.0 V were taken to study the electrochemical reaction mechanism. When a sweep rate of 0.1 mV s^−1^ was applied, peaks located at 1.05 and 0.3 V can be clearly observed in the first cathodic scan, as shown in Fig. 4a. Similar to the electrochemical reaction of other TMDs, the peak at 1.05 V is indexed to the formation of Na_x_ReSe_2_ owing to the insertion of Na^+^ into the ReSe_2_ layer [46, 47]. The next broad peak at 0.3 V is attributed to the SEI formation [48] as well as to the reduction reaction of the transfer of Na_x_ReSe_2_ to Na_2_Se and metallic Re [49]. The reaction mechanism of NIBs can be summarized in accordance with Eqs. 1 and 2, which corresponded to a two-step conversion reaction process. Starting from the second cycle, the positions of these two peaks moved to 0.5 and 1.375 V, respectively. Regarding the anodic scan, an intense peak detected at 1.74 V is relevant to the desodiation of the Re + Na_2_Se composites into Na_*x*_ReSe_2_ and the subsequent formation of ReSe_2_ [50]. Except for the first cycle, the following curves are extremely similar in shape and size in justification of the superior stability of ReSe_2_@G@CNFs during sweeping.

1 $$ {\text{ReSe}}_{2} + x{\text{Na}}^{ + } + x{\text{e}} \to {\text{Na}}_{x} {\text{ReSe}}_{2} $$

2 $$ {\text{Na}}_{x} {\text{ReSe}}_{2} + \left( {4 - x} \right){\text{Na}}^{ + } + \left( {4 - x} \right){\text{e}} \to {\text{Re}} + {\text{Na}}_{2} {\text{Se}} $$

The galvanostatic discharge–charge profiles of ReSe_2_@G@CNFs at the current density of 200 mAh g^−1^ are presented in Fig. 4b. The first discharge/charge capacities are 384/253 mAh g^−1^ and correspond to the relatively low CE of 66%. The main cause of the irreversible capacity loss is the formation of the SEI film [51]. In the subsequent cycles, the coulombic efficiency is nearly 100%. All curves are going to be highly consistent, thus indicating a good reversible capacity. In addition, the charge and discharge voltage platforms are consistent with the CV curves. Figure 4c displays the cyclic performance of ReSe_2_@G@CNFs at 200 mA g^−1^. This shows a stable long-term cycle life yielding 227 mAh g^−1^ after 500 cycle and a capacity at 89% compared to the second discharge capacity. Specifically, the coulombic efficiency is close to 100% during most of the cycles. In contrast, ReSe_2_@CNFs yield a lower specific capacity of 175 mAh g^−1^. This phenomenon results from the regulation of graphene which modifies the structural characteristic of carbon nanofibers. Pure ReSe_2_ was also synthesized (Fig. S8) and exhibited a high initial capacity, but significantly decayed as the cyclic number increased. The measurement of electrical conductivity was completed with the use of the two-probe method. At first, ReSe_2_ carbon fibers mats were cut into rectangular sheets with sizes of 25 × 20 mm^2^. Subsequently, the two ends of the mats were clamped through two metal probes which were connected to the semiconductor parameter analyzer (Agilent 4156). As depicted in Fig. S9, the ReSe_2_@G@CNFs exhibit a linear response with an increased slope and correspond to a smaller resistance compared to ReSe_2_@CNFs. The results show that graphene can effectively improve the conductivity, consequently leading to a higher capacity. Additionally, the cyclic performances of ReSe_2_@G@CNFs (1 mM) were evaluated, which delivered a capacity of 199 mAh g^−1^, while the capacity retention was 81% after 500 cycles (Fig. S10a). Moreover, ReSe_2_@G@CNFs (0.4 mM, Fig. S10c) yielded the minimum specific capacity which was lower than 200 mAh g^−1^ owing to the relatively low content of ReSe_2_. Therefore, the appropriate concentration also had a great impact on the electrochemical performance. The rate capability of the composites was also investigated (Fig. 4d). The outcomes showed that reversible capacities of 283, 241, 197, 170, and 140 mAh g^−1^ were maintained when the current densities, respectively, increased from 100 to 2000 mA g^−1^. Meanwhile, the capacity can return to 223 mAh g^−1^ and continue to cycle without a significant decay as the current density returns to 200 mA g^−1^. Obviously, the rate performances of the ReSe_2_@CNFs were secondary, and they delivered an average capacity of approximately 125 mAh g^−1^ at large current values. In Fig. S10b, d, both ReSe_2_@G@CNFs (1 mM) and ReSe_2_@G@CNFs (0.4 mM) exhibit much lower capacities. Furthermore, at low current density values, the responses exhibit attenuation trends. In general, the outstanding electrochemical property of ReSe_2_@G@CNFs is owing to its specific composition and morphology. Graphene modification enhances its electrical conductivity, while the moderate amount of ReSe_2_ will relieve the agglomeration of nanoparticles. Meanwhile, the structure of nanofibers can effectively alleviate the volume change of the electrode during the sodiation/desodiation process and provide a fast electronic/ionic transmission path.

For better understanding of the kinetics of ReSe_2_@G@CNFs, the electrochemical impedance spectra of the composite before/after 100 cycles were acquired. The Nyquist plot shows a depressed semicircle at high frequencies. This is attributed to the SEI resistance and charge transfer. In addition, the slope of the straight line obtained at low frequencies is attributed to ionic diffusion [52, 53]. As shown in Fig. S11, the size of the semicircle becomes larger after circulation in comparison with previously published results [54–56]. The enlarged resistance may be attributed to the stable SEI films and the phase transition during the conversion reaction process. As the reaction progresses, a large amount of electrolyte is consumed and Na ions continually deposit on the surface of SEI films, thus resulting in the increase in the interface impedance [57–59]. From another perspective, the diffusion rates of ions before and after the completion of the multiple cycles are close in value. This is verified again by the superior electrochemical performance of ReSe_2_@G@CNFs. The EIS of ReSe_2_@CNFs was also conducted and yielded a much larger circle in the medium–high-frequency area compared to those for ReSe_2_@G@CNFs. The result is also in accordance with the *I*–*V* curve. More importantly, as it can be observed from the SEM diagrams of the ReSe_2_@G@CNFs electrodes in Fig. S12a, b, the original smooth fiber surface becomes coarse and thick after 100 cycles, but there is no obvious pulverization in the structure. However, as shown in Fig. S12c, d, the structure of the ReSe_2_@CNF electrode material changed dramatically, as the carbon fibers melted and accumulated into larger particles. This discovery can also explain the outstanding stability of ReSe_2_@G@CNFs.

**Fig. 5:**
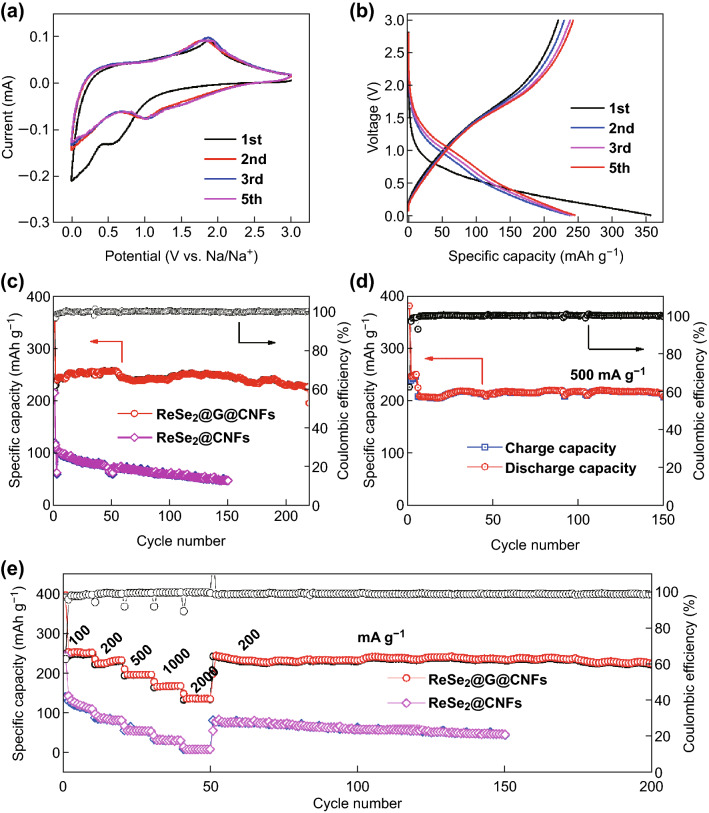
K^+^ storage performances of as-synthesized samples. **a** CV curves corresponding to the initial five cycles. **b** Charge/discharge profiles of ReSe_2_@G@CNFs. **c** Cyclic capability of ReSe_2_@G@CNFs at 200 mA g^−1^. **d** Long-time performance of ReSe_2_@G@CNFs at 500 mA g^−1^. **e** Variation in rate performances of ReSe_2_@G@CNFs in the range from 100 to 2000 mA g^−1^

We also studied the potassium storage performance of ReSe_2_@G@CNFs. The CV curve in Fig. 5a was obtained at a sweep rate of 0.1 mV s^−1^. The electrochemical reactions of KIBs are the same as those for NIBs, and K^+^ was inserted into the layer space of ReSe_2_ to form K_x_ReSe_2_, and then reverted to K_2_Se and metallic Re by a conversion reaction [41, 50]. This process corresponds to a peak of approximately 0.6 V in the first cathodic scan, while its value was approximately 1 V in later scans. During the process of anodic scan, K_2_Se and Re were oxidized into ReSe_2_ and were centered at 1.8 V. Figure 5b shows the first five cycles of the discharge–charge profiles conducted at 200 mA g^−1^. This process delivered the first capacities of 356 and 220 mAh g^−1^ that led to a lower initial CE (62%) compared to NIBs, which can be ascribed to the different electrolyte consumptions for the formation of SEI films, and the different electrochemical properties of the alkali metal selenide (Na_2_Se/K_2_Se). In the second cycle, the charge/discharge curves are similar in shape, thus indicating increased reversibility. The cyclic performance conducted at 200 mA g^−1^ is shown in Fig. 5c. After 200 cycles, ReSe_2_@G@CNFs can maintain the capacity of 230 mAh g^−1^, while the retention value reaches 97% compared to the second discharge, and the CE is close to 100%. Its superior cyclic stability is far greater compared to many other reported results on anodes in KIBs (Table S1). By contrast, the corresponding values for ReSe_2_@CNFs are much lower, yielding rapid capacity degradation during the charge/discharge process. The protective effect of graphene and carbon is more obvious in KIBs. In addition, Fig. 5d presents the cyclic performances of the samples in the presence of large currents of the order of 500 mA g^−1^. It is noted that the first 150 cycles exhibited almost no attenuation in capacity, but there was a slight increase owing to the activation of the electrolyte and active materials as the reaction progressed. Figure 5e depicts the superior rate performance of as-prepared material at various conditions. ReSe_2_@G@CNFs exhibited the capacities of 254 to 157 mAh g^−1^ as the current increased from 100 to 2000 mA g^−1^, respectively. It even maintained a stable capacity of 250 mAh g^−1^ when it recovered to a value of 200 mA g^−1^. The capacity then started to slow down gradually. However, ReSe_2_@CNFs delivered lower specific capacities when different current densities were applied, and this value was close to zero especially for large currents equal to 2 A g^−1^. We also studied the long cyclic performances of ReSe_2_@G@CNFs used as KIB anodes, as shown in Fig. S13, whereby a high discharge capacity of 178 mAh g^−1^ at 200 mA g^−1^ was maintained, and the capacity retention reached 73% after 550 cycles. These results indicate that ReSe_2_@G@CNFs have excellent electrochemical properties.

**Fig. 6:**
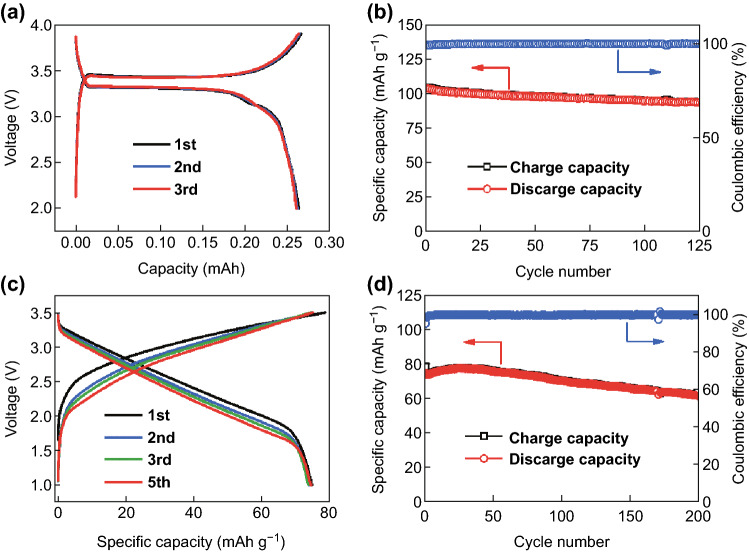
Electrochemical properties of full cells based on Na_3_V_2_(PO_4_)_3_ and ReSe_2_@G@CNFs. **a**, **c** Charge/discharge curves, and **b**, **d** respective cyclic capabilities of NVP cathode and full cells at 1C

**Fig. 7:**
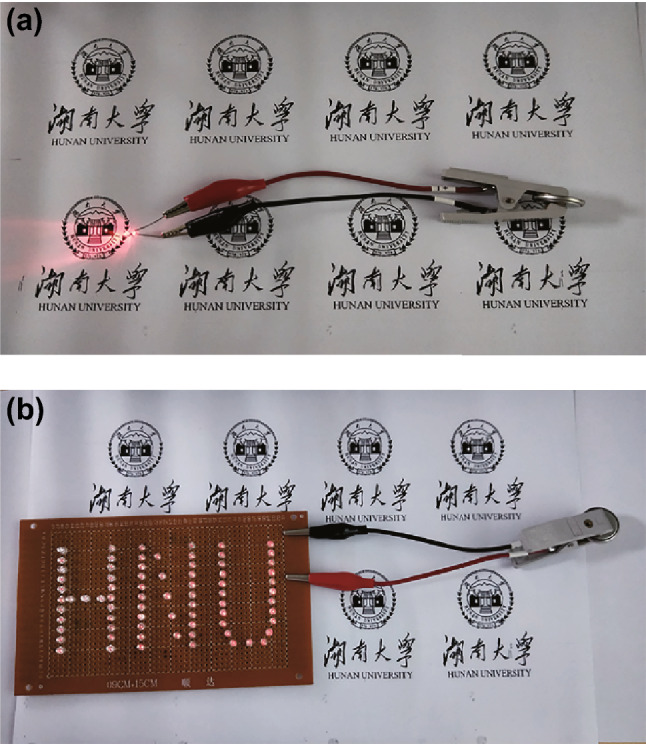
Digital images of ReSe_2_@G@CNFs||NVP full cells showing the lighting pattern of LED bulbs: **a** a single LED and **b** a LED array

It is also essential to investigate the practical applications of ReSe_2_@G@CNFs. Hence, we have successfully assembled Na^+^ full cells by adapting ReSe_2_@G@CNF as the negative electrode and Na_3_V_2_(PO_4_)_3_ as the positive electrode. NVP is a promising cathode for Na^+^ full cells and is known for unique NASICON-type open frameworks, high-operating voltage platforms, and excellent cyclic and rate performances [60]. In this study, the NVP/C mixture was prepared by a ball mill and high-temperature calcination process (Fig. S14), and the XRD plot is shown in Fig. S15. Figure 5b exhibits an initial discharge capacity of 103 mAh g^−1^ at 1C, thus showing a high capacity retention rate of 90% after 125 cycles when applied in half cells. Regarding the full cells of ReSe_2_@G@CNFs||NVP, ReSe_2_@G@CNF anodes usually exert discharge platforms below 1.8 V, while the NVP exerts a 3.3 V discharge platform and a 3.45 V charge platform (Fig. 6a), according to previous results, as shown in Fig. 4b. Therefore, we set the voltage window of full cells in the range between 1 and 3.5 V. As depicted in Fig. 6c, a tilting voltage platform from 3.25 to 1.75 V is obvious during the discharge process in full cells and also delivers a stable discharge capacity. It is worth noting that the capacities of full cells were computed based on the quality of the cathode. In Fig. 6d, the cathode displays a stable discharge capacity of 74 mAh g^−1^ and the capacity was reserved as 82% after 200 cycles. To further verify the practical applications of the sodium-ion full cells, LED lamp experiments were conducted. Figure 7a shows that a single LED bulb is easily lit up by a fully charged button battery. Despite the load on the LED matrix which was arranged to form the word HNU (consisted of 75 small LED bulbs), the button battery could still be powered normally (Fig. 7b). We also tested the brightness time of the LED array, as shown in Fig. S16. The brightness of the array becomes progressively less intense as a function of time and is almost zero after 2 h. These excellent electrochemical performances of the full cells demonstrate the potential applications of ReSe_2_@G@CNFs for energy storage.

## Conclusions

In summary, we have successfully synthesized ReSe_2_–carbon nanofibers through electrospinning and heat treatment. Its advantages lie in the extremely weak van der Waals coupling interaction and large interlayer spacing of ReSe_2_, and shortened diffusion channel for the ion and electron because of carbon nanofibers. Based on reasonable control schemes of the morphology and composition, the ReSe_2_@G@CNFs led to excellent electrochemical performances when used in NIBs and KIBs, even in full cells. These compounds delivered reversible capacities of 227 mAh g^−1^ after 500 cycles in NIBs, 230 mAh g^−1^ at 200 mA g^−1^ after 200 cycles, and 212 mAh g^−1^ at 500 mA g^−1^ after 150 cycles in KIBs. Additionally, they also led to a capacity retention of 82% after 200 cycles in full cells. Most importantly, this was the first time we investigated the battery applications of ReSe_2_ and obtained good results. Based on the study’s findings, we envisage that ReSe_2_ will draw more attention for energy storage applications.

## Electronic supplementary material

Below is the link to the electronic supplementary material.

Supplementary material 1 (PDF 1394 kb)
