# Supplementary material for: Improved Na+/K+ Storage Properties of ReSe2–Carbon Nanofibers Based on Graphene Modifications
Source: Nanomicro Lett. 2019 Mar 11;11:22. doi: 10.1007/s40820-019-0248-2 (PMC7770771; doi:10.1007/s40820-019-0248-2)
Supplement: Supplementary file 1 — Supplementary material 1 (PDF 1394 kb) [file 40820_2019_248_MOESM1_ESM.pdf]

Supporting Information for

Improved Na^+/K^+ Storage Properties of $ReSe_2$-Carbon Nanofibers Based on Graphene Modifications

Yusha Liao[1], Changmiao Chen[1], Dangui Yin[1], Yong Cai[1], Rensheng He[1,]*, Ming Zhang[1,]*

[1]Key Laboratory for Micro/Nano Optoelectronic Devices of Ministry of Education, Hunan Provincial Key Laboratory of Low-Dimensional Structural Physics and Devices, School of Physics and Electronics, Hunan University, Changsha 410082, People's Republic of China

*Corresponding authors. E-mail: zhangming@hnu.edu.cn (Ming Zhang); hdwlhrs@hnu.edu.cn (Rensheng He)

Supplementary Figures and Tables

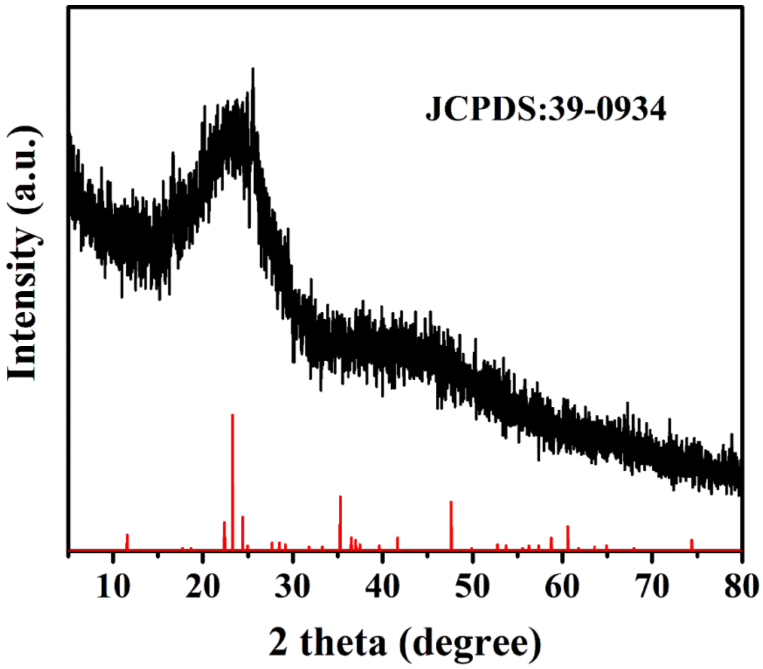

Fig. S1 XRD pattern of Re_2O_7@G@CNFs

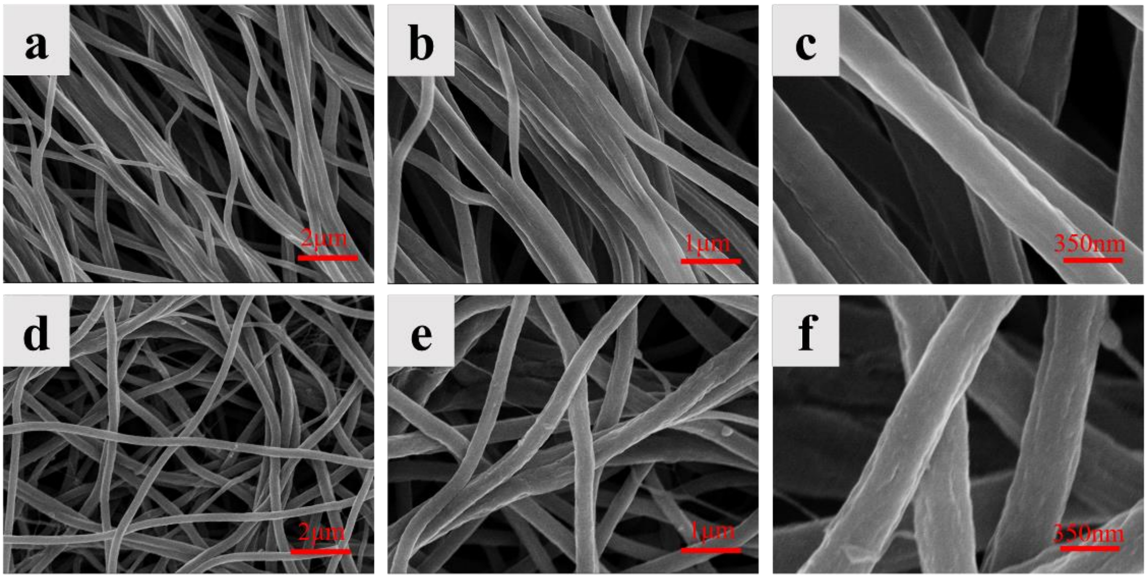

Fig. S2 a-c SEM images of Re_2O_7 CNFs without GO. **d-f** SEM of $ReSe_2$@CNFs

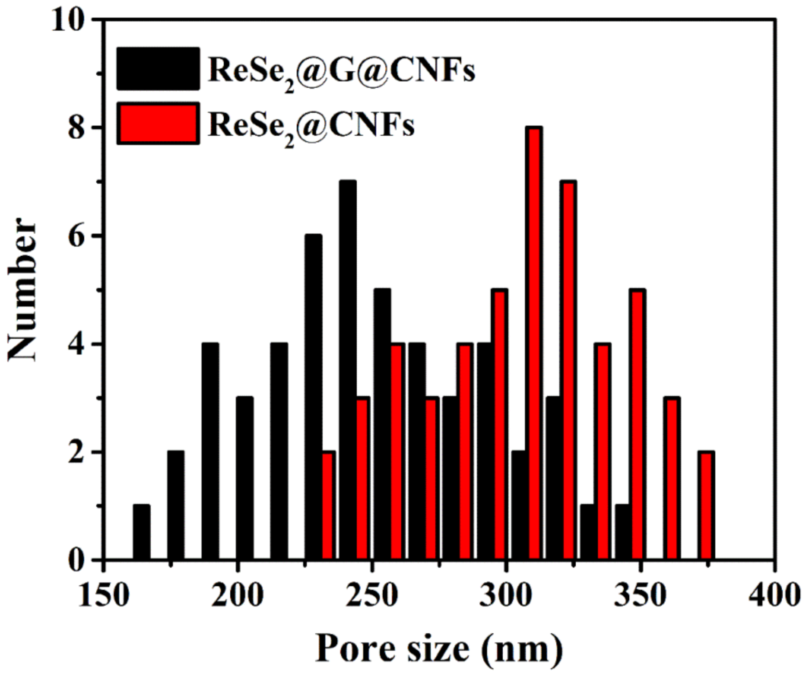

Fig. S3 The diameter distribution of $ReSe_2$@G@CNFs and $ReSe_2$@CNFs. We randomly selected 50 fibers to measure their diameters. According to the statistics, $ReSe_2$@G@CNFs has the highest probability of appearing 240 nanometers in diameter, while 308 nanometers in $ReSe_2$@CNFs

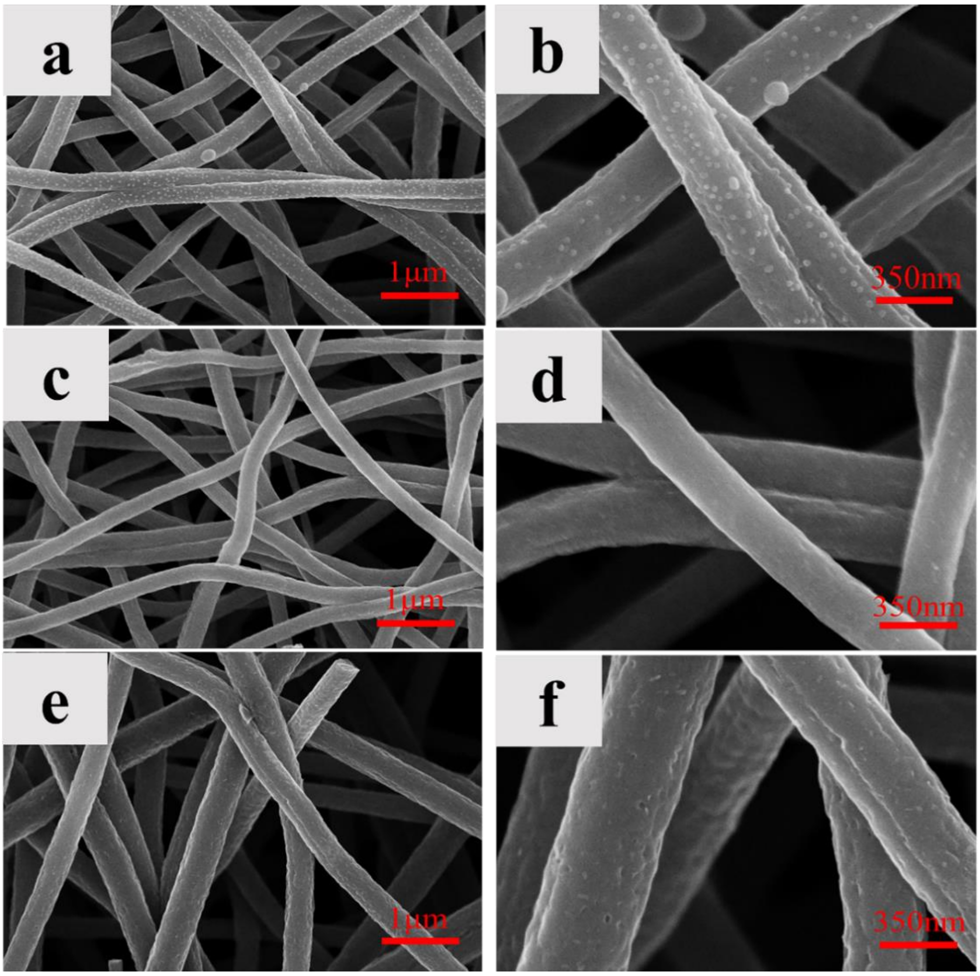

Fig. S4 a-b SEM of Re_2O_7 CNFs with 1mmol Re. **c-d** SEM of 1 mM-$ReSe_2$@G@CNF and **e-f** SEM of 0.4 mM-$ReSe_2$@G@CNFs

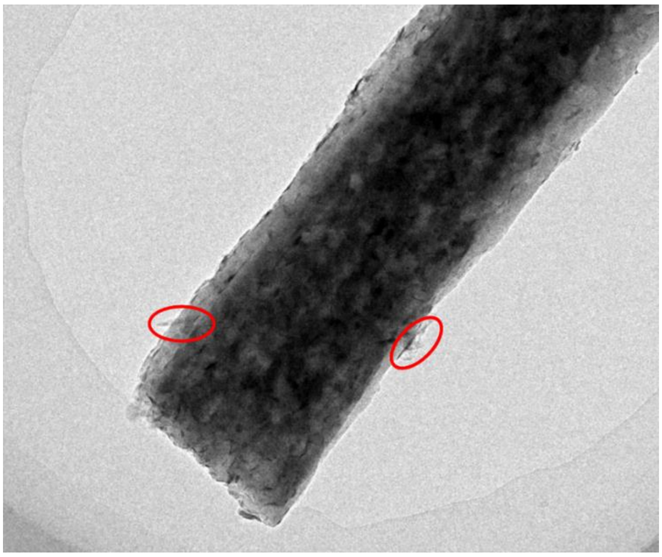

Fig. S5 The TEM image of $ReSe_2$@G@CNFs

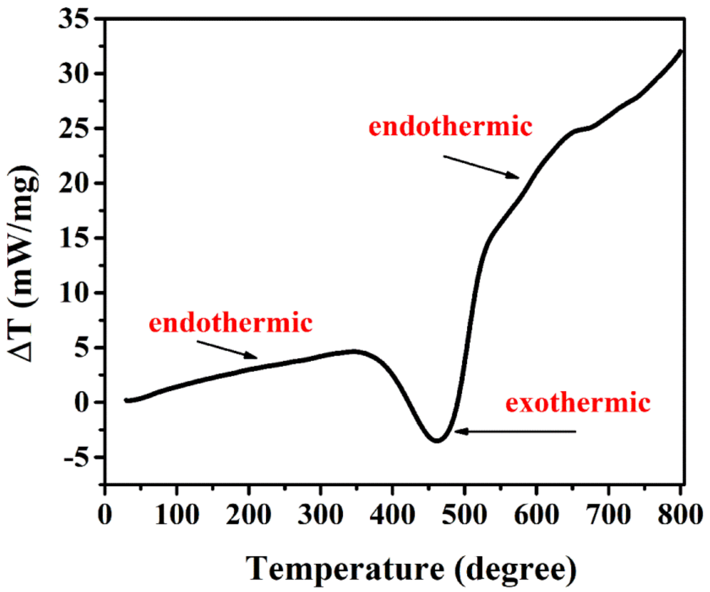

Fig. S6 Differential Thermal Analysis (DTA) plot of $ReSe_2$@G@CNFs. The endothermic peak and exothermic peak correspond to different types of reactions

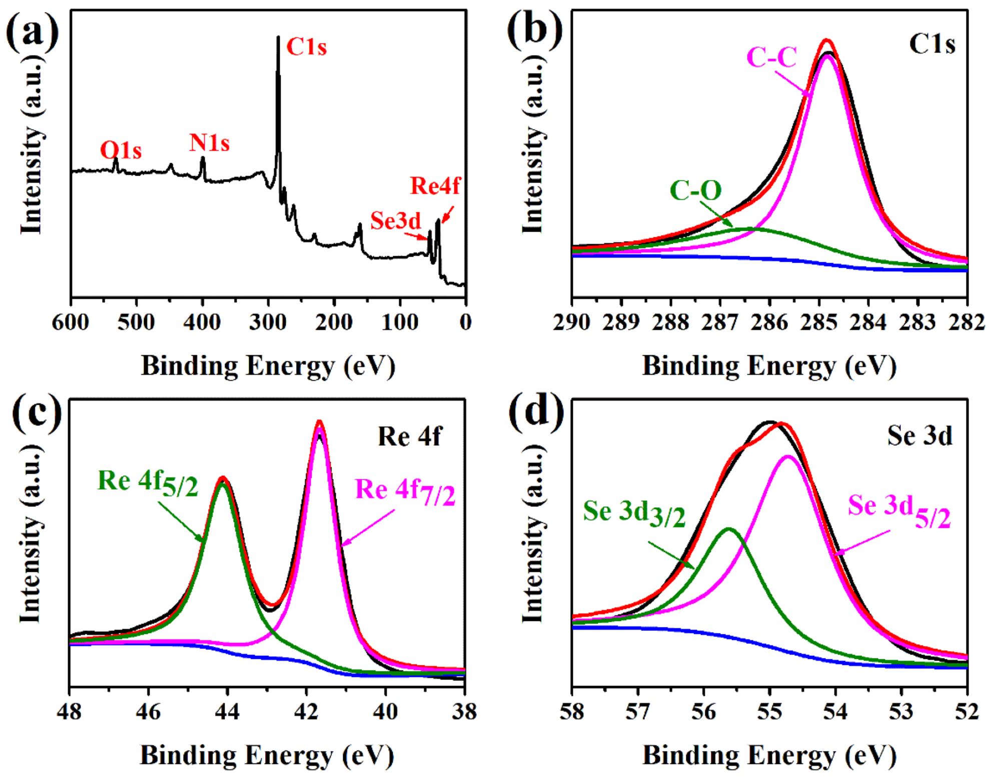

Fig. S7 The XPS analysis of $ReSe_2$@G@CNFs. **a** XPS survey spectrum of $ReSe_2$@G@CNFs, and the high-resolution spectrum of **b** C 1s, **c** Re 4f, and **d** Se 3d

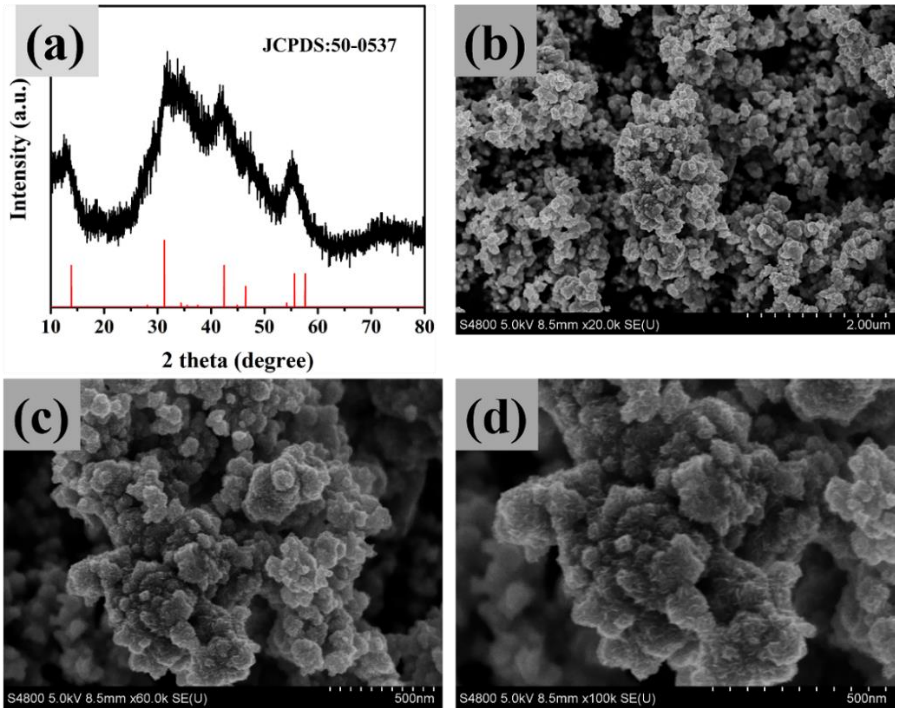

Fig. S8 a XRD and **b-d** SEM of the as-prepared pure $ReSe_2$

The pure $ReSe_2$ was synthesized through hydrothermal method, detailed process as follows: Firstly, 0.26g NH_4ReO_4 and 0.32g Se power were dispersed in ethylene glycol and hydrazine hydrate hybrid solution stirred for half an hour. Then the solution was transferred into a 50ml stainless steel Teflon-lined autoclave and maintained at 200℃ for 24h. After cooling to room temperature, the material was washed with distilled water and ethanol for several times and dried in a vacuum oven overnight. Finally, the collected sample was annealed at 600℃ for 2h in Ar atmosphere. The XRD plot in Fig. S8 is well matched to JPCDS:50-0537, which is verified the existence of $ReSe_2$. From the SEM images, we can see many nanoparticles were consisted of tiny nanoflakes, forming a flower-sphere structure.

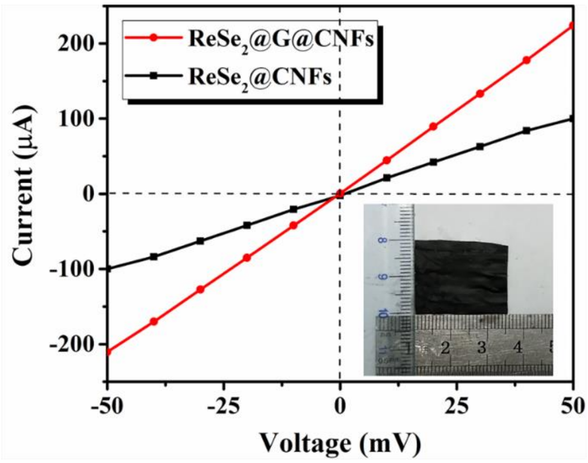

Fig. S9 I-V curves of two samples between -50 to 50 mV

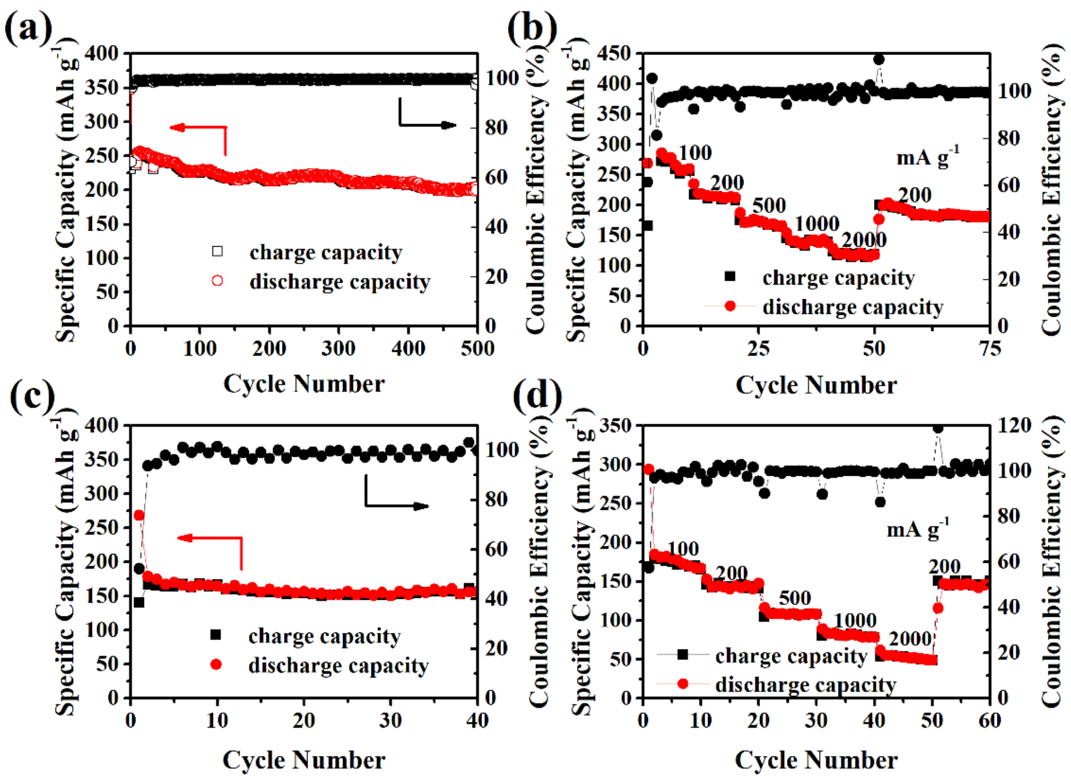

Fig. S10 a, c cycle and **b, d** rate performance of 1 mM-$ReSe_2$@G@CNFs and 0.4 mM-$ReSe_2$@G@CNFs in NIBs, respectively

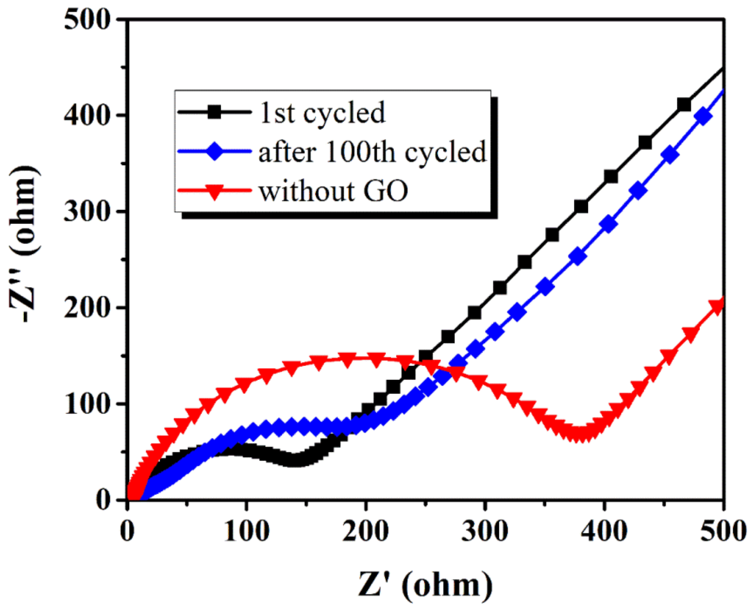

Fig. S11 Nyquist plot of $ReSe_2$@G@CNFs before (black line) and after (blue line) 100 cycles, as well as the EIS of $ReSe_2$@CNFs (red line). The first impedance test was performed after a cycle of CV test

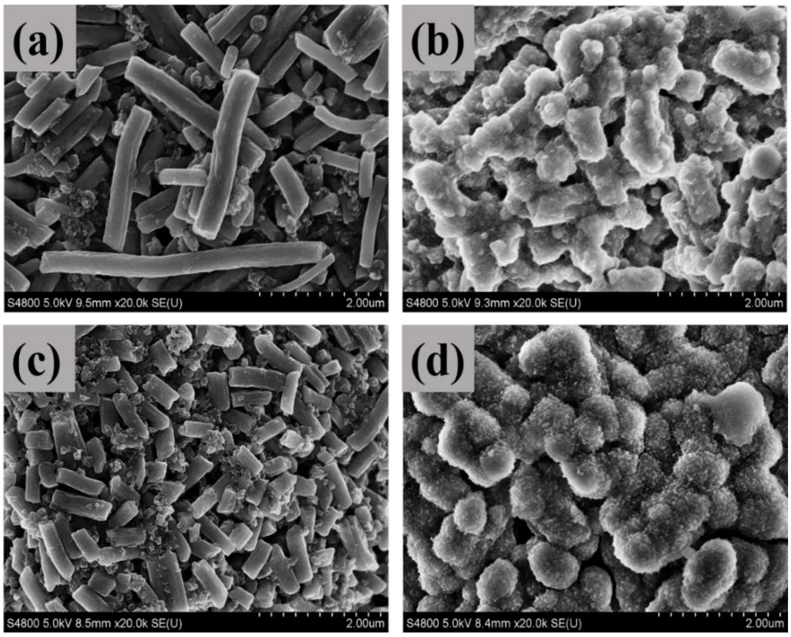

Fig. S12 SEM of **a-b** $ReSe_2$@G@CNFs electrode before/after 100 cycles. **c-d** $ReSe_2$ @CNFs electrode before/after 100 cycles

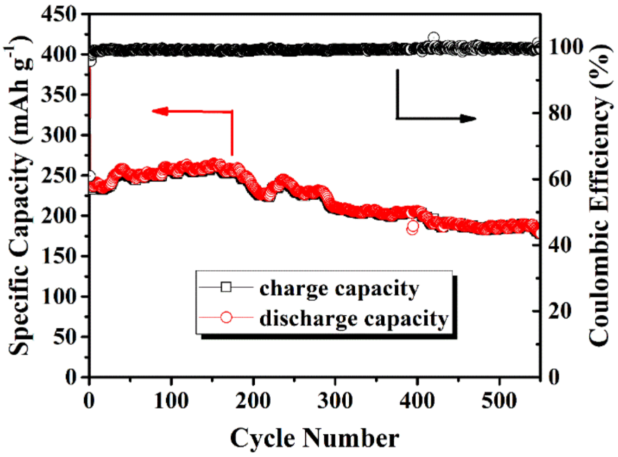

Fig. S13 Long cyclic performance of $ReSe_2$@G@CNFs at 200 mA g^{-1} after 550 cycles in KIBs

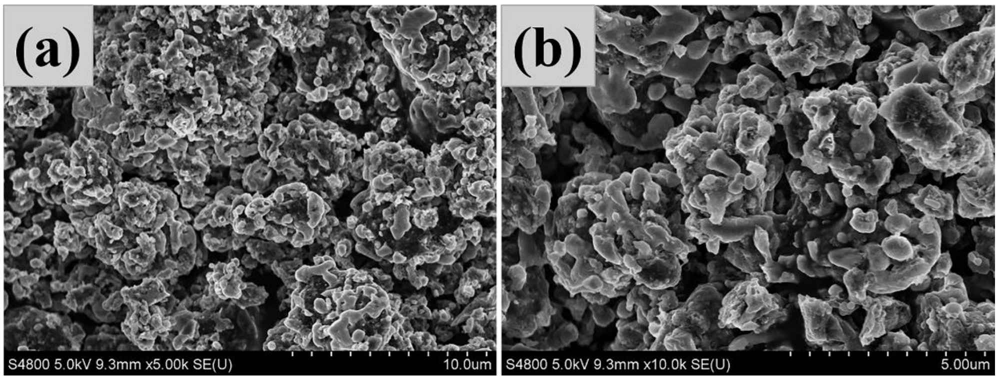

Fig. S14 SEM images of NVP/C composites synthesized by ball milling

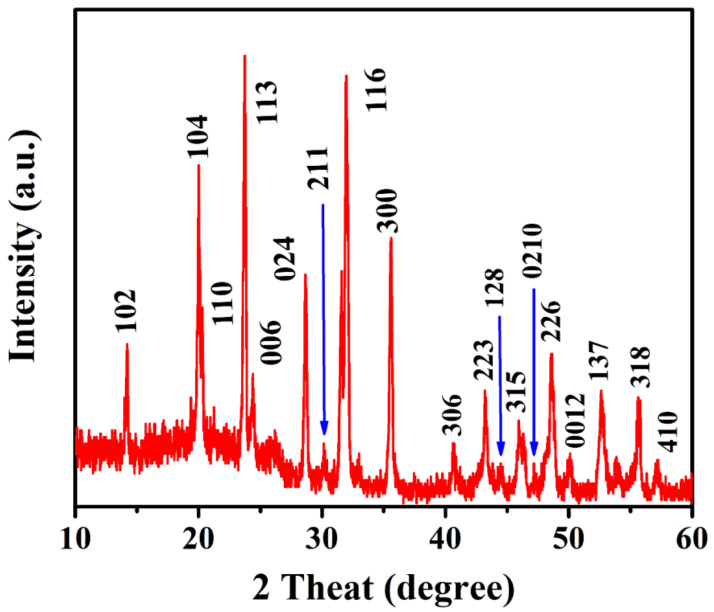

Fig. S15 XRD image of NVP/C composites

The $Na_3V_2(PO4)_3/C$ composites were prepared through a facile ball-milling method. Typically, 21 mmol NaH_2PO_4•$2H_2O$, 14 mmol NH_4VO_3 and 1.26 g PAN were putted in an agate jar, then the jar was ground at a rate of 400 rpm for 12 h. The prepared precursor was annealed in argon/hydrogen gas at 800 ℃ for 8 h.

For the fabrication of full cells, $Na_3V_2(PO_4)_3/C$ mixed with Super P and polyvinylidene fluoride (PVDF) (8:1:1 by weight) was spread on aluminum foil and adopted as the cathode. The loading mass of active material was about 2.3~2.5mg/cm^2 for cathode. The $ReSe_2$ CNFs and $Na_3V_2(PO_4)_3/C$ were assembled in CR2032 coin cells, and the mass ratio of anode to cathode was controlled at about 1:1.8 to balance the capacity.

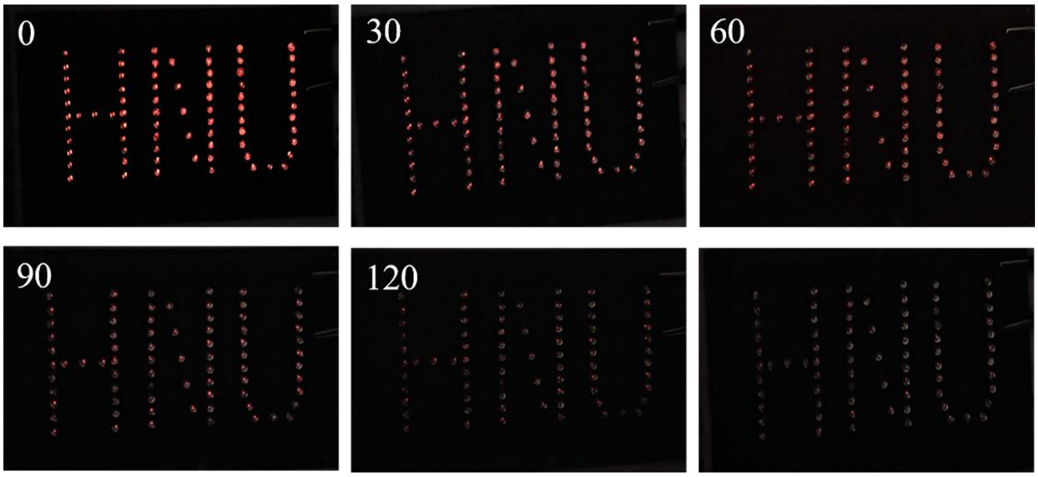

Fig. S16 The LED array lighted for 0, 30, 60, 90, and120 min and it almost out at last

Table S1 The comparison of K^+ storage properties of various anodes

Materials	Voltage Range (V vs. K / K^+)	Capacity (mAh g^{-1}) / Current Density (mA g^{-1}) / Cycles	Rate Capacity (mAh g^{-1}) / Current Density (mA g^{-1})	Capacity Retention / Current Density (mA g^{-1}) / Cycles	Refs.
**Nitrogen-doped graphene**	0.01-1.5	~210/100/100th	200/100; 50/200	78%/100/100th	[1]
**Tin-based composite**	0.01-2	~110/25/30th	\	73%/25/30th	[2]
**$K_2Ti_8O_{17}$**	0.01-3	~110.7/20/50th	80/100; 60/200; 50/400; 44.2/500	\	[3]
**Hard-carbon microspheres (HCS)**	0.01-1.5 1 C=280mA/g	~216/0.1C/100th	262/0.1C; 245/0.2C; 205/1C; 190/2C; 136/5C	83%/0.1C/100th	[4]
**Graphitic materials**	0.01-2	\	270/5; 266/10; 234/50; 141/200	\	[5]
**Hard-soft composites carbon**	0.01-2 1 C=279mA/g	~200/1C/200th	230/0.5C; 210/1C; 190/2C; 121/5C; 81/10C	93%/1C/200th	[6]
**MXene-Derived $K_2Ti_4O_9$**	0.01-3	~88/50/100th	150/20; 119/50; 105/100; 97/150; 89/200; 8/300	61%/50/100th	[7]
**3D porous carbon/Sn composites**	0.01-3	~276.4/50/100th	310/50; 280/100; 200/200; 150/500	70%/50/100th	[8]
**Sn_4P_3/C**	0.01-2	~307.2/50/50th close to 0 after 120th	399.4/50; 221.9/1000	80%/50/50th	[9]
**Nitrogen-rich hard carbon**	0.01-3 1 C=280mA/g	~205/0.12C/200th	250/0.12C;205/0.36C;190/0.72; 180/1.8C; 170/3.6C; 160/7.2C;	\	[10]
**$ReSe_2$@G@CNFs**	0.01-3	~226/200/220th ~178/200/550th ~212/500/150th	254/100; 235/200; 203/500; 182/1000; 157/2000	95%/200/220th 73%/200/550th 86%/500/150th	this work

Supporting References

[1] K. Share, A.P. Cohn, R. Carter, B. Rogers, C.L. Pint, Role of nitrogen doped graphene for improved high capacity potassium ion battery anodes. ACS Nano **10**(10), 9738–9744 (2016). https://doi.org/10.1021/acsnano.6b05998

[2] I. Sultana, T. Ramireddy, M.M. Rahman, Y. Chen, A.M. Glushenkov, Tin-based composite anodes for potassium-ion batteries. Chem. Commun. **52**(59), 9279-9282 (2016). https://doi.org/10.1039/C6CC03649J

[3] J. Han, M. Xu, Y. Niu, G.N. Li, M. Wang, Y. Zhang, M. Jia, C.M. Li, Exploration of $K_2Ti_8O_{17}$ as an anode material for potassium-ion batteries. Chem. Commun. **52**(75), 11274-11276 (2016). https://doi.org/10.1021/acsnano.6b05998

[4] Z. Jian, Z. Xing, C. Bommier, Z. Li, X. Ji, Hard carbon microspheres: Potassium-ion anode versus sodium-ion anode. Adv. Energy Mater. **6**(3), 1501874 (2016). https://doi.org/10.1021/acsnano.6b05998

[5] W. Luo, J. Wan, B. Ozdemir, W. Bao, Y. Chen et al., Potassium ion batteries with graphitic materials. Nano Lett. **15**(11), 7671-7677 (2015). https://doi.org/10.1021/acs.nanolett.5b03667

[6] Z. Jian, S. Hwang, Z. Li, A.S. Hernandez, X. Wang, Z. Xing, D. Su, X. Ji, Hard-soft composite carbon as a long-cycling and high-rate anode for potassium-ion batteries. Adv. Funct. Mater. **27**(26), 1700324 (2017). https://doi.org/10.1002/adfm.201700324

[7] Y. Dong, Z.S. Wu, S. Zheng, X. Wang, J. Qin, S. Wang, X. Shi, X. Bao, Ti_3C_2 MXene-derived sodium/potassium titanate nanoribbons for high-performance sodium/potassium ion batteries with enhanced capacities. ACS Nano **11**(5), 4792-4800 (2017). https://doi.org/10.1021/acsnano.7b01165

[8] K. Huang, Z. Xing, L. Wang, X. Wu, W. Zhao, X. Qi, H. Wang, Z. Ju, Direct synthesis of 3D hierarchically porous carbon/Sn composites via in situ generated nacl crystals as templates for potassium-ion batteries anode. J. Mater. Chem. A **6**(2), 434-442 (2018). https://doi.org/10.1039/C7TA08171E

[9] W. Zhang, J. Mao, S. Li, Z. Chen, Z. Guo, Phosphorus-based alloy materials for advanced potassium-ion battery anode. J. Am. Chem. Soc. **139**(9), 3316-3319 (2017). https://doi.org/10.1021/jacs.6b12185

[10] C. Chen, Z. Wang, B. Zhang, L. Miao, J. Cai et al., Nitrogen-rich hard carbon as a highly durable anode for high-power potassium-ion batteries. Energy Storage Mater. **8**, 161-168 (2017). https://doi.org/10.1016/j.ensm.2017.05.010
